# Analytical Methods for the Identification of Edible and Feed Insects: Focus on DNA-Based Techniques

**DOI:** 10.3390/foods14112002

**Published:** 2025-06-05

**Authors:** Kamila Zdeňková, Eliška Čermáková, Pavel Vejl, Agáta Čermáková, Jakub Vašek

**Affiliations:** 1 Department of Biochemistry and Microbiology, University of Chemistry and Technology Prague, Technická 5, 166 28 Prague, Czech Republic; kamila.zdenkova@vscht.cz; 2 Department of Food Science, Czech Agrifood Research Center, Drnovská 507/73, 161 00 Prague, Czech Republic; 3 Department of Genetics and Breeding, Faculty of Agrobiology Food and Natural Resources, Czech University of Life Sciences Prague, Kamýcká 129, 165 00 Prague, Czech Republic; vejl@af.czu.cz (P.V.); cermakovaagata@af.czu.cz (A.Č.); vasek@af.czu.cz (J.V.)

**Keywords:** insect species identification, edible insect, PCR, DNA barcoding, sequencing

## Abstract

The utilization of insects as a source of essential nutrients holds considerable promise, with the potential to serve as both feed and food. Consequently, there is a necessity to develop control systems, as the undeclared addition of insects to food products and/or non-compliance with labelling regulations may pose health risks and result in financial losses for consumers. This review describes methods for identifying and detecting insect species by targeting biomolecules such as DNA, proteins, saccharides, and metabolites, with a particular focus on DNA-based approaches. This review provides a detailed overview of the application of polymerase chain reaction (PCR) and DNA sequencing methods that are suitable for the analysis of edible and forage insects. The main focus is on identifying species that are approved for use as novel foods or insect feeds within the European Union (e.g., house cricket (*Acheta domesticus*), common mealworm (*Tenebrio molitor*), migratory locust (*Locusta migratoria*), lesser mealworm (*Alphitobius diaperinus*), black soldier fly (*Hermetia illucens*), banded cricket (*Gryllodes sigillatus*), field cricket (*Gryllus assimilis*), silkworm (*Bombyx mori*)). However, insect species of global relevance are also discussed. The suitability of DNA analysis methods for accurate species identification, detection of (un)labeled contaminants, and monitoring of genetic diversity has been demonstrated.

## 1. The Emerging Role of Edible Insect Species in Food and Feed

Insects are a common food source in many subtropical countries, primarily due to their high protein content. With the growing demand for sustainable and nutritious food, the popularity of insects as food is slowly increasing in Europe as well. This has led to a surge in insect products on the European market, such as roasted insects or chips, often made from crickets, mealworms, and grasshoppers. Despite growing interest in edible insects as a sustainable source of protein, their acceptance in Western societies remains limited due to concerns about allergenicity, cultural aversions, low awareness, and lack of knowledge. The acceptance of insects as food is shaped by factors such as cultural norms, sensory qualities, perceived health benefits, affordability, availability, and environmental impact. Insects are generally more readily accepted when used as feed for aquaculture, livestock, or pets—particularly in the form of live insects, untreated or processed whole insects (e.g., freeze-dried), or extracted insect-based proteins and lipids [1,2,3,4].

Insect-based products can be prepared in a number of ways, including roasting, drying, milling into flour, or isolating selected components such as proteins or lipids for addition to food. However, in the EU, insects are considered novel foods under Regulation 2015/2283, which means that their marketing is subject to strict legislation. The authorization process involves a scientific safety assessment by the European Food Safety Authority (EFSA) and takes at least 17 months [4]. A simplified procedure is possible for foods traditionally consumed in third countries, provided their safety is documented [5]. In early 2025, six insect products from four species, house cricket (*Acheta domesticus*), common mealworm (*Tenebrio molitor*), migratory locust (*Locusta migratoria*), and lesser mealworm (*Alphitobius diaperinus*), have been approved as novel foods, with further applications being evaluated [1,2,3]. The use of insect proteins in animal feed is also regulated by the EU. Proteins, fat, or whole insect bodies from species such as the black soldier fly (*Hermetia illucens*), common mealworm, common housefly (*Musca domestica*), house cricket, banded cricket (*Gryllodes sigillatus*), field cricket (*Gryllus assimilis*), or silkworm (*Bombyx mori*) may be used, for example, in aquaculture, poultry feed, and pig feed, whereas their use as feed for ruminants remains prohibited. Furthermore, appropriate substrates for insect farming are strictly regulated to ensure food and feed safety.

Despite the potential benefits of insects as a food and feed source, such as high protein content, favorable amino acid profiles, and the potential immunostimulant and antimicrobial properties of chitin (a polysaccharide found in insect exoskeletons), there are notable challenges that need to be addressed. A major concern is the risk of pathogen transmission, as insects can carry pathogenic organisms, including bacteria, parasites, micromycetes, and yeasts. Insects can act as mechanical or biological vectors of pathogens if they are not adequately starved or if the breeding hygiene is poor [6]; in the natural environment, insects can also act as intermediate hosts or mechanical vectors of parasites. The consumption of unprocessed insects may therefore be a significant risk factor, and therefore effective processing operations and hygiene principles should be implemented to minimize the risk of contamination with foodborne pathogens [7,8,9,10]. Grabowski et al., (2017) [11] demonstrated that the microbiological profile varies by product type. Dried and powdered insects contain significantly higher total bacterial counts than fried and cooked insects. For example, *B. cereus*, coliforms, *Serratia liquefaciens*, *Listeria ivanovii*, *Mucor* spp., *Aspergillus* spp., *Penicillium* spp., and *Cryptococcus neoformans* were detected [11]. Ensuring the microbiological safety of edible insects, often through heat treatment, is crucial for food safety and shelf life. However, the microbial risks associated with edible insects are considered to be comparable to other animal protein sources. Certain insect proteins (e.g., tropomyosin, arginine kinase) may cause allergic reactions, particularly in individuals already allergic to shellfish, mites, or crustaceans. Insect feed allergens such as gluten may also persist in the final product. These allergenic risks can be mitigated using processing methods like hydrolysis or fermentation [9,12,13,14].

The edible insect market may be susceptible to fraud due to the adulteration risks, including species substitution, the use of unauthorized or non-breeding (wild-caught) insects, and improper breeding practices. Processing insects into unrecognizable forms, such as flour or meal, allows replacing the insect portion with a cheaper (e.g., plant) raw material [2,4]. As edible insect products become increasingly available on the market, developing reliable analytical methods to verify insect species and their feed substrates is essential [1,2]. This review summarizes the methodologies currently used to detect insects intended for human consumption or animal feed. The first section provides an overview of these methods categorized by their target analytes, including macromolecules and metabolites. The second section focuses specifically on DNA-based detection techniques, particularly PCR and DNA barcoding.

## 2. Insect Species Authentication Techniques for Food and Feed Applications

Based on the type of target analyte, the method can be classified as analyzing proteins/peptides, saccharides, other metabolites, and nucleic acids. Each of these categories offers specific advantages and is suitable for different analytical purposes, depending on the requirements of the specific application, the time available, and the resources of the laboratory, whether financial, human, or technological. In addition to these targeted analyses, methods that focus on the detection of insect bodies or parts thereof by microscopic analysis are also used (Table 1). For example, a protocol for the isolation and detection of insect material in aquatic feeds, based on sedimentation with non-polar solvents to concentrate insect body particles, combined with light microscopy, successfully identified insect body fragments of *H. illucens*, *T. molitor*, *G. assimilis*, and *A. diaperinus* [15]. Histochemical methods are also used; e.g., *A. domesticus* and *T. molitor* were successfully detected in snack bars [16].

### 2.1. Proteins as Analytical Targets

Techniques for protein identification include methods such as mass spectrometry (e.g., LC-MS, matrix-assisted laser desorption/ionization time-of-flight mass spectrometry: MALDI-TOF MS) and electrophoresis, which allow both targeted analysis of specific proteins and non-targeted fingerprinting of overall protein profiles; MS techniques are increasingly being used for the specific detection of trace amounts of proteins and peptides in food. These molecules are relatively stable during processing, although analysis may require complex protein separation techniques. Crucial to improving the accuracy of detection is the expanding database of edible insect proteins and peptides [17]. In work by Francis et al. [18], proteomic analysis was performed on four edible insect species—*T. molitor*, *H. illucens*, *A. diaperinus* and *G. assimilis*—by LC-MS. Although the analysis yielded reproducible results, identification efficiency varied among species. *T. molitor* and *G. assimilis* were accurately identified, whereas *H. illucens* could not be reliably identified [17,18,19].

Antigens can include various chemical structures, most commonly proteins, including glycoproteins or lipoproteins, and polysaccharides, which are often detected using immunochemical methods like enzyme-linked immunosorbent assay (ELISA) and immunoblotting. In a study by Karnaneedi et al. [20], sodium dodecyl-sulfate polyacrylamide gel electrophoresis (SDS-PAGE) and immunoblotting with allergen-specific antibodies and sera from crustacean-allergic patients were employed to monitor potential allergens in two insect species, house cricket (*A. domesticus*) and black soldier fly (*H. illucens*). The study revealed cross-reactivity of shrimp-specific antibodies to tropomyosin from both insect species, indicating significant sequence and structural similarities between shrimp and insects. Additionally, unique allergens were identified in both species, including hemocyanin, vitellogenin, HSP20, apolipophorin-III, and chitin-binding protein, highlighting their allergenic potential [20].

Immunochemical tests are mainly used to compare the allergenicity of edible insect species in Asia with known allergens. They are used less frequently for specific analyses of individual species to identify unique allergens, as has been done for silkworms. Jeong et al. [21] identified a 27 kDa hemolymph glycoprotein as a heat-stable IgE-binding component in serum samples from patients allergic to silkworm. Similarly, Liu et al. [13] used western blot and ELISA to identify *B. mori* arginine kinase as a major allergen, with sera from all ten patients tested reacting to a 42 kDa protein and the crude silkworm extract. Furthermore, *B. mori* arginine kinase shows cross-reactivity with cockroach arginine kinase. However, further large-scale studies are required to assess the wider applicability of this approach [13,21]. Commercial ELISA kits for insect detection are still few on the global market, with the most available assays targeting specific insect pests or allergens, such as bed bugs (*Cimicidae*) or mites (*Acari*).

### 2.2. Polysaccharide Targets in Insect Detection

Recently, innovative methods have been developed for the specific detection of N-acetylglucosamine in chitin and chitosan polymers from selected insect species in food. One such approach uses an indirect sandwich enzyme-linked lectin sorbent assay (ELLA) with wheat germ agglutinin (WGA), which is highly specific for N-acetylglucosamine [22]. In addition, the carbohydrate composition of mealworm larvae was successfully analyzed using HPLC [23].

### 2.3. Metabolites

Metabolite analysis focuses on the identification of small molecules (<1500 Da) characteristic of specific species or substrates. Metabolomics and non-targeted screening (NTS) have emerged as powerful tools in food science, relying heavily on advanced analytical platforms such as gas chromatography (GC) and liquid chromatography (LC) coupled to high-resolution mass spectrometry (HRMS). These techniques allow both targeted and untargeted analysis, often generating molecular patterns without necessarily identifying or quantifying specific compounds. They are valuable for authenticating insect species, monitoring the use of prohibited feed substrates (e.g., animal by-products), and detecting contaminants or residues in insect-derived products. While these methods are currently being developed for use in the detection of edible insects, they have the potential to be used in the future, for example as a screening method, due to their high-throughput capacity [2,38].

Poma et al. [2] discussed the use of metabolomics and NTS in traditional food systems and explored strategies to adapt and implement entometabolomics—the application of metabolomics and NTS to edible insects—into food analysis. Direct analysis in real-time high-resolution mass spectrometry (DART-HRMS) was used to distinguish four insect species, each with a unique metabolic fingerprint. *B. mori* was characterized by a high abundance of linolenic and quinic acids, whereas *H. illucens* showed a predominance of palmitic and oleic acids. Chemometric analysis further revealed that proline was a key discriminating molecule for *T. molitor*, while palmitic and linoleic acids were the most informative molecular features for *A. domesticus* [2,24].

### 2.4. DNA as Detection Marker

DNA-based methods, including PCR, isothermal amplification, and advanced techniques such as next-generation sequencing (NGS), provided high specificity and accuracy. These methods can be used to uniquely identify insect species or detect non-targeted species. Their high sensitivity and accuracy make them ideal for regulatory control and food and feed safety purposes. The choice of the appropriate method depends on the specific requirements of the analysis—for example, whether the objective is routine screening or more in-depth research analysis. Combinations of several techniques may also be used to achieve the most accurate and reliable results.

In general, nucleic acid-based approaches offer a broader range of verified protocols suitable for routine analysis. Therefore, the following section provides a detailed comparison of the advantages and limitations of DNA-based insect analysis for species identification and detection of potential adulteration in edible insect products. Regulatory frameworks often require precise species identification to prevent the inclusion of unauthorized, allergenic, or non-native species, which could pose health risks or violate trade laws. Reliable and validated molecular tools based on target DNA analysis, such as precise PCR amplification or DNA barcoding analysis including sequencing, are therefore important for supporting regulatory enforcement, facilitating market transparency, and ensuring consumer protection.

## 3. Insect DNA Analysis

**Figure 1:**
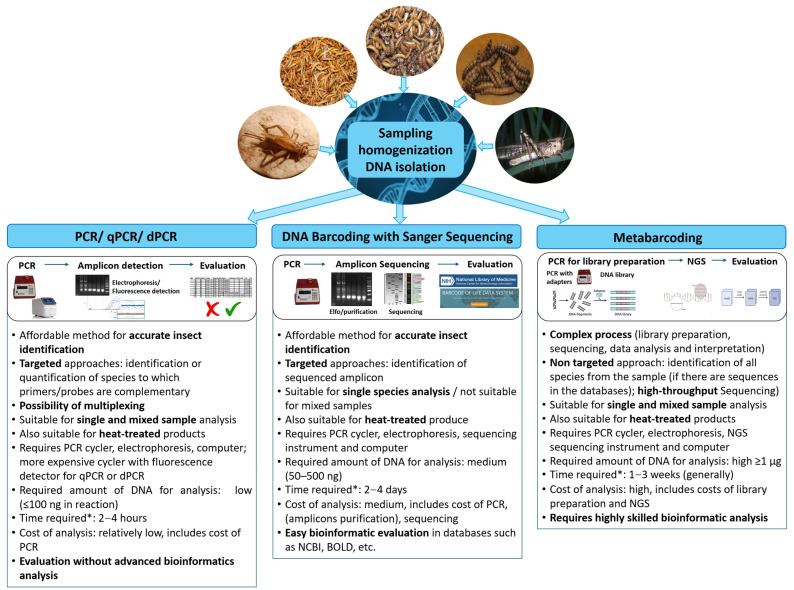
Comparison of approaches for analyzing edible and foraging insect DNA, *: time required for analysis itself and subsequent analysis.

Currently, PCR amplification of specific DNA fragments is commonly used for food authentication. In addition, sequencing technologies are gaining popularity, including Sanger sequencing of selected amplicons (i.e., DNA barcoding) and high-throughput massive parallel sequencing of all DNA in a sample (metabarcoding) [25,26,35,38]. A general comparison of used approaches for analyzing edible and feed insect DNA is shown in Figure 1 and discussed in detail below.

### 3.1. Advances in PCR Techniques for Analysis of Insect DNA

All three generations of PCR—conventional end-point PCR (first generation), quantitative real-time PCR (qPCR; second generation), and digital PCR (dPCR; third generation)—are currently applied, either in singleplex or multiplex formats, for the detection of insect species in food and feed products [27,39,40,41,42]. A comparative summary of these PCR methods and protocols applied in insect DNA analysis is presented in Table 2. PCR-based techniques target both mitochondrial (mtDNA) and nuclear (nDNA) genetic markers for the authentication of food and feed insect species, utilizing established and validated protocols.

The mitochondrial markers used are cytochrome b (cyt b) for *A. domesticus* [27]; cytochrome oxidase I (COI) for *H. illucens* [32], *A. diaperinus* [25,28], *A. domesticus* [26], *B. mori* [29], *Galleria mellonella* [25], *Gryllus bimaculatus* [29], *T. molitor* [29], *Oxya chinensis* [29], larvae of *Protaetia brevitarsis* and *Allomyrina dichotoma* [29], *L. migratoria* [26], *Zophobas atratus* [25], and unspecified insects [26]; NADH dehydrogenase for *T. molitor* [26], *Apis cerana*, *A. dorsata*, and *A. mellifera* [30]; and 16S rDNA for insects [35], *A. domesticus* [25], *B. mori* [25], *G. sigillatus* [25], *L. migratoria* [25], *Schistocerca gregaria* [25], and *T. molitor* [25]. Regarding nDNA markers, the gene encoding cadherin was used for *T. molitor* [43], *A. diaperinus* [31], and *B. mori* [44], as well as *wingless* for *T. molitor* [43] and 18S rDNA [43]. Selecting an appropriate DNA marker is challenging, especially for highly processed matrices or quantitative applications. Shorter amplicons enable PCR analysis of processed foods (where DNA is fragmented), and mtDNA markers are generally preferred due to their higher copy number. However, while mtDNA offers higher sensitivity and specificity, its variable copy number limits its reliability for quantitative analyses [39,45,46,47].

qPCR remains the preferred method for food and species authentication thanks to its high levels of specificity, sensitivity, and reproducibility. Nevertheless, accurate quantification of insect content in food or feed using qPCR or dPCR is challenging, as results are typically expressed as DNA-to-DNA ratios, which do not directly correlate with the actual mass fraction of the target species. For example, determining the amount of cricket in pasta made with cricket flour requires converting DNA-based measurements into mass-to-mass ratios. To enable such conversions, the use of reference mixtures with known composition (e.g., gravimetrically defined mass ratios) is essential during method development to establish reliable correction factors. While such reference materials are well established for GMO quantification and are available for some meat-based products (e.g., sausages), certified reference materials for insect-based foods are currently lacking.

### 3.2. Insect DNA Barcoding

DNA barcoding is a molecular technique used to identify and differentiate species based on knowledge of the primary sequence of a short, standardized region of their DNA. The selected DNA region is amplified by PCR, followed by sequencing and comparison of the primary sequence with National Center for Biotechnology Information (NCBI) or Barcode of Life Data System (BOLD) database [48,49,50]. The aim of the technique, known as standard DNA barcoding, is to identify a specific organism based on the primary DNA sequence of a characteristic specific genomic region (the so-called ‘DNA barcode’) and typically involves Sanger sequencing. In contrast, metabarcoding aims to identify entire communities of organisms present in a sample by analyzing the total DNA extracted.

While it follows the same basic principle of knowing the primary sequence of the DNA barcoding region, metabarcoding involves the simultaneous analysis of many species. A conserved genomic region is amplified by PCR and then sequenced by NGS, allowing high-throughput identification of multiple taxa within a single sample. Metabarcoding thus allows characterization of many biological species present in a given sample [35,51].

#### 3.2.1. Insect DNA Barcoding with Sanger Sequencing

DNA barcoding with Sanger sequencing is widely used in the food industry, e.g., to identify the source of material in fish products, vertebrate products, food products, and ready meals in restaurants [36,39,52]. For example, the COI sequence was successfully used for monitoring of samples from market, which were amplified and sequenced using a variety of primer combinations, and the obtained sequences were compared with reference sequences in databases (GenBank, BOLD) in the work of Siozios et al. [36]. Further, different COI fragments (approximately 760 bp in length) were used for differentiation between *A. diaperinus* and *Alphitobius laevigatus* [31]. The question is whether fragments of this length would be successfully detected in processed food.

Mitochondrial markers are very useful for barcoding because of the smaller size of mtDNA (i.e., higher stability in technological processes used in food preparation), its circular structure, and its higher abundance in cells. However, the limitation is still the small number of annotated insect reference sequences available in databases, although recent progress has been rapid, and sequences are constantly being added. In addition, detection of DNA in processed food or feed is better when targets shorter than 250 bp are used [43,53,54]. DNA barcoding using mitochondrial markers has also been tested and shown to be effective for the identification of insect pests. This sequencing approach was applied to representatives of 19 insect orders comprising 191 species selected from pest lists compiled by institutions involved in food safety and pest control research [33]. A combination of two COI markers and a 16S rRNA marker, each less than 200 base pairs in length, was used. These short markers allowed successful species identification even in samples subjected to heat treatment at 118 °C for 18 min. To assess the practical performance of the method, 38 insect species from seven taxonomic orders—Diptera, Psocoptera, Blattodea, Coleoptera, Hemiptera, Thysanoptera, and Lepidoptera—were tested, successfully detecting insects used as feed, such as the common housefly. DNA from all samples was successfully amplified and sequenced, and the resulting DNA barcode identifications matched the morphological identifications in 89% of cases. In a few cases, DNA sequence identity fell below 95%, mainly due to the lack of matching reference sequences in public databases such as GenBank. One notable example was a sample of black carpet beetle *(Attagenus unicolor japonicus*), which matched oriental silverfish (*Ctenolepisma villosa*) with 97% identity. This discrepancy was probably due to a mislabeled entry in the GenBank database; the sequence in question may actually belong to an *Attagenus* species [33].

This makes it challenging to select a sequence for DNA barcoding, which is conserved in the primer-binding region but sufficiently variable in the sequence bounded by these primers to be used for accurate species identification. Another limitation is the inappropriateness of this method for the analysis of multi-species samples. For this reason, real-time PCR remains the reference technique for species identification in food and feed [36,39,43,53].

DNA barcoding can be used effectively when species-specific PCR systems fail to amplify the target sequence, but non-specific systems (targeting another DNA area) confirm the presence of insect DNA. In such cases, an amplicon derived from an unknown species can be directly sequenced or subjected to additional PCR amplification using barcode primers. A wide range of barcode primers is available in the literature, allowing for the selection of specific segments and amplicon lengths. Longer amplicons generally improve species identification accuracy; however, in technologically processed foods and feeds, extensive DNA fragmentation may necessitate the amplification of shorter fragments. In cases when DNA of only one species is the template for the obtained amplicon, a BLAST search can accurately identify the corresponding insect species [26,39].

Despite significant advancements in methods for the analysis of edible and feed insects, the number of detectable species remains limited due to the restricted availability of suitable detection techniques. Additionally, for many non-approved insect species, even those with known DNA sequences in databases, reliable PCR-based detection methods are lacking.

#### 3.2.2. Insect DNA Metabarcoding

DNA metabarcoding, using massive parallel sequencing, offers a key advantage by enabling the simultaneous identification of multiple insect species with a single analytical approach [26,55]. In a study by Hillinger et al. [35], DNA metabarcoding was employed using a 200 bp fragment of mitochondrial 16S rDNA, which was sequenced on Illumina platforms. This approach proved effective in distinguishing over 1000 insect species. Furthermore, a novel mtDNA universal primer pair was developed for a singleplex PCR assay, enhancing the accuracy and applicability of insect detection [35]. Guisti et al. [34] used metabarcoding of a 200 bp region of the 16S rRNA gene to authenticate 46 processed insect-based products (IBPs). The analysis revealed a high mislabeling rate (33%), influenced by the e-commerce platform and insect species, particularly *A. domesticus*. Partial substitution of high-value species with lower-value species was also detected, as well as the presence of insect pests.

A limiting factor in the widespread application of metabarcoding methods is the lack of sequencing equipment in many laboratories, as well as insufficient computational resources required for processing large datasets. Additionally, there is a shortage of qualified bioinformaticians, and there are gaps in sequence databases, particularly for insect species. By integrating various applications in one sequencing run, such as joint sequencing of plants, animals, or bacteria, the costs of NGS analysis can be substantially reduced. This strategy could encourage more laboratories, particularly those that routinely analyze larger number of samples, to invest in sequencing equipment and adopt metabarcoding techniques [35]. In addition to conducting sequencing at analytical or research laboratories, outsourcing sequencing to specialized, accredited companies is commonly employed. When outsourcing sequencing services to these specialized facilities, it is essential to ensure the security of shared data and thoroughly verify the entire process, both within the laboratory and at the outsourcing company, prior to issuing a sample analysis protocol. However, these concerns can be addressed, and many laboratories successfully rely on the services of specialized sequencing providers.

Amplicon metabarcoding, which involves the high-throughput sequencing of taxonomically informative genetic markers such as the COI or 16S rRNA genes, has become a widely used approach for determining species composition in complex biological samples, including insect-based foods [56,57]. Despite its popularity, this technique is prone to biases introduced during the PCR amplification step. These PCR biases stem from variations in primer binding affinity and template availability, resulting in non-uniform amplification of target sequences. Consequently, this can distort species representation, particularly in samples containing both abundant and rare taxa. Elbrecht and Leese (2015) clearly demonstrated that using universal primers in metabarcoding often leads to the overrepresentation of certain taxa, compromising accurate quantification and even detection of minor species [58].

#### 3.2.3. Beyond PCR Bias: Whole-Genome Sequencing as an Alternative to Amplicon-Based Metabarcoding

An alternative approach is whole genome metagenomic sequencing (WGS metagenomics), also known as shotgun metagenomics, which enables direct sequencing of all DNA present in a sample without the need for prior PCR amplification. This untargeted approach significantly reduces PCR-related artefacts and allows for a more accurate representation of the entire taxonomic diversity, including both abundant and low-frequency species. Importantly, WGS metagenomics is not limited to pre-defined barcoding loci, providing a broader scope for detecting diverse taxa from mixed matrices.

The usefulness of WGS metagenomics for detecting insects has already been demonstrated. For example, Garrido-Sanz et al., (2023) used this approach with food samples and successfully identified 18 insect species, including *Drosophila* spp., from bulk DNA alone, without the need for specific marker genes [37]. Furthermore, integrating real-time base calling into nanopore sequencing enables rapid species identification, providing a promising tool for food authentication and traceability.

Despite its technical advantages, WGS metagenomics has limitations. These include higher operational costs, increased computational demands, and the requirement of substantial input DNA, which can pose a challenge when analyzing processed foods. Furthermore, reduced sensitivity in detecting rare taxa is a consequence of sequencing depth constraints and the overwhelming presence of dominant DNA. Nevertheless, rapid advancements in sequencing technology, including cost reductions and the use of AI-assisted bioinformatic pipelines, are expected to make WGS metagenomics more accessible and practical for routine species identification in the near future.

## 4. Practical Approaches to Monitoring Insect Product Authenticity

As the number of authorized species and their specific developmental stages increases, robust identification methods are becoming essential in order to prevent food fraud and ensure that only approved insect products enter the market. There have been several documented cases of species misidentification, such as unauthorized species being offered as novel foods on the EU market (*B. mori*) or errors in the labelling of commercially sold mealworms [26,31,35]. Examples of mislabeling include the identification of specimens of *A. laevigatus* as *A. diaperinus* [31]. In 2022, inspectors from the State Veterinary Administration found 6.5 kg of frozen Asian silkworm larvae in a Prague warehouse supplying Asian grocery stores and restaurants. As silkworm larvae are not included on the EU’s list of approved insect species for sale as novel foods, they cannot be legally marketed or consumed within the EU. Furthermore, the product lacked the labelling required by food safety regulations. This was the first seizure of this kind under veterinary supervision in the Czech Republic to be recorded [59].

Advances in molecular methods such as metabarcoding have further highlighted the risk of misclassification of insects. Giusti et al. [34] found an error rate of around 30% in their dataset; this was confusion of the accepted species *A. domesticus* with another cricket species, such as crazy red cricket (*G. locorojo*). As the analyses were performed using bioinformatic processing of genetic sequences, the primary confounding species was identified as *G. locorojo*, which, thanks to Weissman et al. [60], is considered to be the correct scientific name for the banana cricket (Jamaican field cricket), which is bred in Europe under the erroneous scientific name *G. assimilis*. These findings highlight the need for comprehensive molecular analyses to characterize the genetic diversity of crickets for food and feed production in Europe.

An interesting example of adulteration of insect products is the mixing of honeys produced by different bee species—in particular, honey from the eastern honey bee (*Apis cerana*) and the giant honey bee (*A. dorsata*), which can be several times more expensive than honey from the western honey bee (*A. mellifera*). This price difference creates opportunities for fraud in the market, such as mislabeling or mixing of honeys of different entomological origins. A PCR test targeting mitochondrial DNA, as proposed in [30], was able to detect as little as 1% admixed *A. mellifera* honey.

In addition to confusion between insects in a sample, the labelling of such products in the EU is not yet precise. Spatola et al. [3] investigated the e-commerce landscape of IBPs in the EU, focusing on food business operators and compliance with EU labelling requirements. Among the 656 IBPs identified, whole insects were the most common (more than 50%), followed by protein products and insect powders, with *A. domesticus* being the dominant species. Regretfully, only 3.4% of IBPs fully complied with EU labelling requirements, with the main problems being missing or inaccurate allergen declarations and other mandatory labelling elements. Therefore, the accidental or deliberate addition of insect ingredients to foods remains a risk for allergy sufferers. Improvements in analytical methods and stricter regulatory controls are therefore expected to lead to appropriate food labelling.

## 5. Current Trends and Future Perspectives

The approval process for insect species as novel foods and for feed applications is constantly evolving, and it is expected that an increasing number of species and their specific developmental stages will be approved in the future. In animal nutrition, the use of insect proteins as a sustainable nutrient source is also expected to increase.

Therefore, there is a need to improve methods for detection of food and feed fraud. The developed methods described in the previous sections can be also used for food safety control by detecting the presence of undesirable insect pests in food, such as the presence of *T. molitor* imago in flour. The near future of edible insect DNA control analysis will continue to be the use of PCR to verify insect species, due to its simplicity, the availability of equipment in many analytical laboratories, the relative ease of interpretation of the results, and the ability to quickly transfer protocols relatively easily between laboratories. Continuous advances in this area allow for faster and better-quality analyses. However, massive parallel sequencing techniques will be increasingly utilized due to their non-targeted screening capabilities and scalability. Improved authentication and traceability methods incorporate some sorts of NGS-like, real-time technologies, such as the MinION nanopore sequencer, which can enable species verification in situ, for example on farms.

The continued supplementing and expansion of genomic reference databases of food and forage insect species will further improve the accuracy of species identification. The increase in annotated primary sequences of insect genomes will also allow for an increase in the accuracy of short sequence alignments of different individuals and genotypes. With the technological progression of individual sequencing platforms, for example, increasing read depth, prediction of single point mutations (SNPs) will also improve; this will also lead to an increase in the reliability of species identification and facilitation of the selection of suitable markers for their identification.

Artificial intelligence and machine learning will play a critical role in automating bioinformatics analysis; bioinformatics tools will be more user-friendly, and artificial intelligence will facilitate data evaluation. In addition, the cost of sequencing has fallen in recent years; given these developments, it is expected that the application of DNA metabarcoding and massive sequencing will be more and more utilized for authentication of insects and food products containing them.

Technological advancements have led to a decrease in the cost of DNA analysis, particularly when processing large numbers of samples. Consequently, it is difficult to compare the costs of implementing PCR versus sequencing-based approaches. Generally, PCR remains the most cost-effective option for directly identifying specific insect species, since numerous verified protocols are available. As PCR also serves as the initial step in DNA barcoding and amplicon-based metabarcoding, it is likely to remain the cheapest method, given that sequencing inherently incurs additional analytical costs.

However, sequencing-based methods offer a broader range of information about the analyzed sample. Unlike PCR alone, which typically confirms the presence of a specific amplicon or its match to a fluorescently labelled probe, sequencing can provide detailed insights into the overall composition of a complex food or feed sample.

Non-targeted analyses, such as DNA metabarcoding, can simultaneously detect multiple species and reveal the full biodiversity within a sample. Therefore, in some cases, this approach may be more economical and informative than running multiple targeted PCR assays. However, the increased use of non-targeted sequencing analyses in control and scientific laboratories is still limited by gaps in sequence databases, a shortage of qualified bioinformaticians, and the lack of reliable, user-friendly software for routine controls. The choice of method should be guided by the specific analytical goal, taking into account the balance between cost and information gained. This decision should be made on a case-by-case basis, depending on the laboratory’s objectives, the complexity of the sample, and the available resources.

The multi-omics approach integrates data from various scientific disciplines, such as genomics, transcriptomics, proteomics, and metabolomics. This provides a comprehensive understanding of complex biological systems, such as complete food and feed products. When applied in the context of veterinary and food safety inspections, multi-omics tools can not only facilitate accurate species identification, but also facilitate the assessment of product quality, safety, and regulatory compliance. These advanced analytical methods could contribute to quicker and more reliable control and monitoring of edible insects and insect-derived feed, which could finally lead to increased consumer belief in and acceptance of edible insects in their food.

## 6. Conclusions

Edible insects are gaining increasing attention as an ecological, nutritionally valuable food source. However, as the demand for insect-based products rises, the risk of food adulteration, especially concerning the authenticity and species identification of insect ingredients, is likely to increase. Therefore, reliable and efficient methods for insect species identification are essential. The selection of appropriate techniques is critical for accurate and effective analysis. DNA-based methods, particularly those leveraging PCR and sequencing technologies, offer high specificity and sensitivity, making them the preferred tools for species identification and quality control in edible insect products. Advances in molecular techniques such as metabarcoding have enabled the detection of unexpected species (not just insects), thus enabling deeper food control. As the costs of sequencing continue to decline, and methodologies improve, integration of DNA analysis into the insect food industry is expected to expand, contributing to more sustainable and reliable food sources in the future. While these methods are already highly effective, there is still room for improvement in terms of their cost-effectiveness, speed, and accessibility in laboratories worldwide. Nonetheless, considering the need for precise species identification and quantification, PCR-based methods remain the ‘gold standard’ for ensuring the authenticity and safety of insect-derived food products.

## Figures and Tables

**Table 1:** Methods used for the detection and identification of edible and feed insects.

Target	Method
Insect body or its parts	Microscopy (light microscope [15])
	Histochemical methods: visualization after staining [16]
Protein	Mass spectrometry (e.g., liquid chromatography mass spectrometry: LC-MS, matrix-assisted laser desorption/ionization time-of-flight mass spectrometry: MALDI-TOF MS) [17,18,19]
	Electrophoresis (SDS-PAGE [20])
	Immunochemical tests (enzyme-linked immunosorbent assay: ELISA, western blot [20,21])
Saccharide	Enzyme-linked lectin sorbent assay (ELLA) [22]
	High-performance liquid chromatography (HPLC) [23]
Metabolites	Gas chromatography (GC) and liquid chromatography (LC) coupled to high-resolution mass spectrometry (HRMS) [2]
	Direct analysis in real-time high-resolution mass spectrometry (DART-HRMS) [2,24]
DNA	PCR and its variations (multiplex PCR, nested PCR, ultrafast PCR system based on a microfluidic chip) [25]
	PCR with fluorescence detection in real-time (qPCR) [26,27,28,29,30,31,32]
	Digital (droplet) PCR (dPCR or ddPCR) [27,31,32]
	Sequencing (Sanger and next-generation sequencing) [33,34,35,36,37]

**Table 2:** Comparison of PCR methods of all three generations and protocols published for edible and feed insect DNA analyses.

Parameters		Endpoint PCR	qPCR with Intercalating Dye	qPCR with Fluorescently Labelled Probe(s)	Digital PCR
Device capacity		Most often 96 reactions/run	Most often 96 reactions/run	Most often 96 reactions/run	Depends on device (8–96)
Price of the device		Low	Medium	Medium	High
Cost of analysis (includes chemicals and plastic only)		Low	Medium	High	Very high
Time required for PCR analysis	Analysis	~5 h *	~3.5 h (Tm analysis)	~2 h	cdPCR: ~2 h; ddPCR: ~3.5 h
	Evaluation	Short	Medium	Medium	Medium
Post-PCR processing		Horizontal agarose electrophoresis	Melt curve analysis	No	Chip/droplet fluorescence reading
Suitable for the analysis of single species samples		Yes	Yes	Yes	Yes
Suitable for the analysis of mixed samples		Yes	Limits in multiplex arrangements	Yes	Yes, with fluorescently labelled probe(s)
Applicability for quantification		No	Yes, single-species samples	Yes	Yes
Specificity		Medium	Medium, limits in multiplexes	High **	High **
Results evaluation requirements		Simple	Moderate	Moderate	Simple
Published protocols for insects		*A. diaperinus* [25], *A. domesticus* [25], *B. mori* [25], *G. mellonella* [25], *G. sigillatus* [25], *L. migratoria* [25], *S. gregaria* [25], *T. molitor* [25], *Z. atratus* [25]	*A. dichotoma* [29], *Apis cerana*, *A. dorsata* and *A. mellifera* [30], *B. mori* [29], *G bimaculatus* [29], *O. chinensis* [29], *P. brevitarsis* [29], *T. molitor* [29], unspecified insects [26]	*A. diaperinus* [28,31], *A. domesticus* [26,27], *H. illucens* [32], *T. molitor* [26,43], *L. migratoria* [26], insects [43]	*A. diaperinus* [31], *A. domesticus* [27], *H. illucens* [32]

* With the inclusion of electrophoresis. ** By using an additional sequence complementary to the target.

## Abbreviations

The following abbreviations are used in this manuscript:

BOLD	Barcode of Life Data System
cdPCR	Capillary digital polymerase chain reaction
COI	Cytochrome oxidase I
Cq	Quantification cycle
cyt b	Cytochrome b
DART-HRMS	Direct analysis in real time high-resolution mass spectrometry
ddPCR	Droplet digital polymerase chain reaction
dPCR	Digital polymerase chain reaction
EFSA	European Food Safety Authority
ELISA	Enzyme-linked immunosorbent assay
ELLA	Enzyme-linked lectin sorbent assay
GC	Gas chromatography
GMO	Genetically modified organism
HPLC	High-performance liquid chromatography
HRMS	High-resolution mass spectrometry
IBP	Insect-based product
LC	Liquid chromatography
LC-MS	Liquid chromatography mass spectrometry
MALDI-TOF MS	Matrix-assisted laser desorption/ionization time-of-flight mass spectrometry
mPCR	Multiplex polymerase chain reaction
MS	Mass spectrometry
mtDNA	Mitochondrial DNA
NCBI	National Center for Biotechnology Information
nDNA	Nuclear DNA
NTS	Non-targeted screening
PCR	Polymerase chain reaction
qPCR	Quantitative polymerase chain reaction
SDS PAGE	Sodium dodecyl sulfate polyacrylamide gel electrophoresis
WGA	Wheat germ agglutinin
UDG	Uracil-DNA glycosylase
WGS	Whole-genome sequencing (and metagenomics)
